# c-kit^+^AT_2_R^+^ Bone Marrow Mononuclear Cell Subset Is a Superior Subset for Cardiac Protection after Myocardial Infarction

**DOI:** 10.1155/2016/4913515

**Published:** 2016-06-27

**Authors:** Mingjun Du, Sebastian Schmull, Wentian Zhang, Chenxi Wang, Feng Lian, Yao Chen, Song Xue

**Affiliations:** ^1^Department of Cardiovascular Surgery, Renji Hospital, School of Medicine, Shanghai Jiao Tong University, No. 160 Pu-Jian Road, Shanghai 200127, China; ^2^Renji-Med X Clinical Stem Cell Research Center, Renji Hospital, School of Medicine, Shanghai Jiao Tong University, No. 160 Pu-Jian Road, Shanghai 200127, China

## Abstract

Although the bone marrow mononuclear cell (BMMNC) is known as an ideal cell type for cell-based therapy for MI treatment, the effective subpopulation still remains unknown. Our study aimed at identifying the optimal subset of BMMNCs suited for cardiac regeneration. In this study, we observed that MI led to (i) a significant increase of the c-kit^+^AT_2_R^+^ BMMNC subpopulation in mice and (ii) a modest increase of AT_2_R^+^ BMMNCs in humans. c-kit^+^AT_2_R^+^ and c-kit^+^AT_2_R^−^ BMMNC subpopulations were obtained from mice after MI. Then, we cocultured cardiac H9C2 cells with c-kit^+^AT_2_R^+^, c-kit^+^AT_2_R^−^, and unfractionated BMMNCs; finally, we found that the c-kit^+^AT_2_R^+^ subset is superior to the c-kit^+^AT_2_R^−^ subset in improving cardiomyocyte protection in vitro. Of note, c-kit^+^AT_2_R^+^ BMMNCs showed a more robust migration capacity than c-kit^+^AT_2_R^−^ and unfractionated BMMNCs* in vitro* and* in vivo*. Additionally, compared to c-kit^+^AT_2_R^−^ and unfractionated BMMNCs, intravenous transplantation of c-kit^+^AT_2_R^+^ BMMNC resulted in smaller infarct size and lower levels of inflammatory reactions in heart tissue, leading to a higher global heart function improvement. In conclusion, our results indicate that the c-kit^+^AT_2_R^+^ BMMNC subpopulation exerts a protective effect against MI and shows promising therapeutic possibilities with regard to the treatment of ischemic heart disease.

## 1. Introduction

Myocardial infarction (MI), commonly known as heart attack, is the most common cause of heart injury and usually results in loss of cardiomyocytes, cardiac dysfunction, and even heart failure [1, 2]. To date, cell transplantation-based regenerative therapy, including the bone marrow mononuclear cell (BMMNC) population, provided us with a promising approach for MI [3, 4]. BMMNCs are a heterogeneous population composed of hematopoietic stem cells (HSCs), mesenchymal stem cells (MSCs), endothelial progenitor cells (EPCs), and many other mature and immature cell types [5]. Due to their comparatively easy isolation and absence of ethical concerns, BMMNCs have become the most commonly used cell source as treatment option [6, 7]. However, despite the advantages mentioned above, the details which BMMNCs exert the protective effects in MI still remain unknown.

c-kit (CD117), a receptor tyrosine kinase, is an important cell surface marker used to identify certain types of hematopoietic progenitors in the bone marrow (BM). Multipotent stem cells derived from BM or myocardium can express c-kit. Plenty of evidence indicates that, after MI, transplantation of undifferentiated stem cells, such as c-kit^+^ BM precursor cells, leads to recovery of cardiac function [6]. Moreover, some studies claim that bone marrow c-kit^+^ cells cause an improved cardiac function independent of transdifferentiation into either cardiac muscle or endothelial cells but are rather associated with cardiac release of cytokines and neovascularization [6]. Nevertheless, some researchers argue that c-kit may promote inflammatory reaction and thus limited the therapeutic use of c-kit^+^ stem cells [8, 9]. Even though there are many debates regarding the underlying mechanism, there is no doubt that c-kit^+^ stem cells exert a positive effect on cardiac repair.

Following MI, the RAS interferes with inflammation and cardiac remodeling processes. Ang II is the main effector peptide of the RAS, which has two receptors, including the AT_1_R and AT_2_R [10]. AT_1_R is ubiquitous and abundant in adult tissues and responsible for most of the detrimental cardiovascular effects of Ang II [11]. The AT_2_R belongs to the 7-transmembrane G-protein coupled receptor family and shares 34% homology with AT_1_R. Additionally, AT_2_R is only highly expressed in fetal tissue and its expression decreases dramatically after birth; but, importantly, it can be reexpressed under pathological conditions without age limit, such as MI and stroke. A protective role of AT_2_R has been shown in tissue repair and regeneration, such as in brain and kidney [12]. In the heart, the assumption that AT_2_R is involved in cardiac regeneration is based on its reexpression during pathological conditions. Mechanistically, the cardioprotective effect of AT_2_R was mediated via the NO pathway, and overexpression of AT_2_R was associated with improved contractile function in adjacent noninfarcted myocardium [13, 14].

Different types of AT_2_R^+^ cells have been used as cell therapy for cardiac regeneration in recent years. Skorska et al. have identified CD4^+^ AT_2_R^+^ T cells in circulating blood of rats, as well as in the postinfarcted heart and spleen. Characterization of CD4^+^ AT_2_R^+^ T cells showed that they expressed anti-inflammatory-related cytokines such as IL-10 and FoxP3. Furthermore, intramyocardial injection of MI-induced splenic CD4^+^ AT_2_R^+^ T cells into recipient rats with MI led to a reduced infarct size and improved cardiac performance [15]. Altarche-Xifró et al. observed that cardiac c-kit^+^AT_2_R^+^ cells increased in response to acute cardiac injury. These cardiac c-kit^+^AT_2_R^+^ cells were characterized by upregulated transcription factors implicated in cardiogenic differentiation and genes required for self-renewal. Moreover, AT_2_R stimulation* in vitro* inhibited apoptosis of cardiomyocytes.* In vivo* AT_2_R stimulation led to an increased c-kit^+^AT_2_R^+^ cell population in the infarcted myocardium and reduced apoptosis of cardiomyocytes in rats with acute myocardial infarction [16]. However, the therapeutic use of AT_2_R^+^ BM-derived stem cells remains illusive.

In this study, we showed that AT_2_R expression of BMMNCs increased and cell numbers of c-kit^+^AT_2_R^+^ BMMNC subpopulation were upregulated after MI. Moreover, we explored a serious potential of c-kit^+^AT_2_R^+^ subpopulation isolated from BMMNCs including antiapoptosis, homing capacity, cytokine secretion, inflammatory repression, and ameliorating global heart function. We demonstrated for the first time that c-kit^+^AT_2_R^+^ BMMNCs are superior to both c-kit^+^AT_2_R^−^ BMMNCs and unfractionated BMMNCs for cardiac repair after MI. All these results may pave the road for future studies and eventually for therapeutic use of the c-kit^+^AT_2_R^+^ BMMNC subpopulation.

## 2. Materials and Methods

### 2.1. Animals

C57BL/6 mice were obtained from the Slac Laboratory Animal Company (Shanghai, China). Animals were maintained in pathogen-free facilities with water and commercial mice food available ad libitum. All experiments have been approved by Shanghai Ren Ji Hospital Ethics Committee and were performed in accordance with ethical standards.

### 2.2. MI Mouse Model

MI induction was performed as follows: mice were anesthetized by mask inhalation of 1.5% isoflurane in supine position. Subsequently, an incision was made at the fourth rib and the heart was exposed. A 7-0 sterile surgical suture was used to ligature the left coronary artery. Hereafter, incisions were closed and wounds were cleaned and disinfected.

### 2.3. Cell Isolation and Flow Cytometry Analysis of Bone Marrow Mononuclear Cells

BMMNCs were isolated at day 7 after MI from mice bone marrow tissue by density gradient centrifugation. In brief, femurs and tibia were harvested from C57BL/6 mice. Bone marrow was collected by repeated washing of the bone marrow cavity with Hanks (Biowest, France) and then loaded on Ficoll solution (ShenZhen DaKeWei Biological Manufacture, China). For gradient centrifugation, cells were centrifuged at 400 ×g for 20 min. Subsequently, the cell layer was isolated; three times the volume Hanks (Biowest, France) was added and centrifuged at 1000 rpm for 5 min. Hereafter, cells were incubated with unlabeled rabbit anti-AT_2_R (1 : 100; Abcam Ltd., HK) and PE-conjugated mouse anti-c-kit (1 : 100; BD Biosciences, Germany) for 30 min at 4°C in the dark. Cells were washed, indirectly labeled with anti-rabbit secondary antibody (Alexa Fluor® 647; Life Technologies, USA) for 30 min at 4°C in the dark, and subjected to flow cytometry. Analysis and cell acquisition were performed on a FACSCalibur cytometer or sorting (c-kit^+^AT_2_R^+^, c-kit^+^AT_2_R^−^, and unfractionated BMMNCs) on BD Accuri FACSAria. Data were analyzed using BD Accuri C6 flow cytometer.

### 2.4. Human Bone Marrow Tissues

The protocol was approved by the ethical committee of Ren Ji Hospital, and written informed consent was obtained from all patients. A total of 10 bone marrow tissues were collected from patients undergoing CABG operation (CABG patients) between January 2014 and June 2014. Furthermore, we also collected bone marrow specimens from patients undergoing aortic valve replacement (other patients; *n* = 10) who had no ischemic heart disease. Bone marrow tissues were aspirated from sternum by using 20 mL syringe before the operation started. Collected bone marrow was mixed 1 : 1 with heparin and transferred to a 15 mL centrifuge tube.

### 2.5. Flow Cytometry Analysis of Human Bone Marrow Mononuclear Cells

Ten times the collected bone marrow volume DMEM was added to the bone marrow-heparin mix and then loaded on Ficoll solution (Biowest, France). For gradient centrifugation, cells were centrifuged at 400 ×g for 30 min. Subsequently, the cell layer was isolated and three times the volume DMEM was added and centrifuged at 1000 rpm for 5 min. Hereafter, cells were incubated with unlabeled rabbit anti-AT_2_R (1 : 100; Abcam Ltd., HK) for 30 min at 4°C in the dark. Cells were washed, indirectly labeled with anti-rabbit secondary antibody (Alexa Fluor 647; Life Technologies) for 30 min at 4°C in the dark, and subjected to flow cytometry. Data were analyzed using BD Accuri C6 flow cytometer.

### 2.6. Coculture Experiments and Determination of Apoptosis of Cardiac H9C2 Cell Line

BMMNC subsets were either seeded in 24-well plates for single culture (5 × 10^5^/well) or seeded in transwell membrane plates of 0.4 *μ*m (Corning Incorporated Life Sciences, USA) and cocultured with cardiac H9C2 cells (kindly provided by Gu Jianming, RenJi Hospital, China; 1 : 5 ratio of BMMNC subset/H9C2 cells). Single or coculture system was incubated in serum-free medium and placed in a hypoxia incubator with 1% oxygen for 48 hours at 37°C. Following incubation, the cells were washed twice in PBS and an apoptosis detection kit (Vitazyme, China) was applied to quantify apoptosis of cardiac H9C2 cells and BMMNC subsets. Cells were analyzed using BD Accuri C6 flow cytometer.

### 2.7. Protein Cytokine Antibody Array

Supernatants of single cultured BMMNC subsets (c-kit^+^AT_2_R^+^, c-kit^+^AT_2_R^−^, and unfractionated BMMNCs) were collected and analyzed using cytokine antibody array (Mouse XL Cytokine Array Kit; R&D, USA) according to manufacturer's instructions. Semiquantitative analysis of the spots was performed with image analysis system (QuantityOne).

### 2.8. Migration Assay* In Vitro*

*In vitro*, transwell migration assays were performed using millicell cell culture inserts (Corning Incorporated Life Sciences, USA) with 8.0 *μ*m pore polycarbonate membranes coated with Matrixgel (1 : 5 mixed with DMEM, BD Biosciences, USA). Briefly, BMMNC subsets were seeded in transwell membrane plates at density of 5 × 10^4^ cells per well. Medium obtained from postinfarct myocyte cultures for 3 days was added to the lower site of transwell migration assays and an incubation step was performed for 24 hours. Subsequently, migrated cells from the lower chamber were collected, resuspended in 100 *μ*L PBS, and finally counted using BD Accuri C6 flow cytometer. A graphical overview is shown in Figure 4(a).

### 2.9. Cell Transplantation

After induction of MI in mice, 1 × 10^6^ of each BMMNC subpopulation were suspended in 200 *μ*L PBS after DiIC18(5) solid staining process [17] and directly injected into the caudal vein. MI control mice received the same volume of single PBS injection. The experimental groups were divided into 4 groups (*n* = 6 for each group): (1) PBS group (control); (2) c-kit^+^AT_2_R^+^ BMMNC group; (3) c-kit^+^AT_2_R^−^ BMMNC group; (4) unfractionated BMMNC group. Cells were allowed to migrate* in vivo* for 7 days.

### 2.10. Cell Tracking* In Vivo*

DiIC18(5) solid (DID) was used to stain BMMNC subsets according to manufacturer's instructions. After preparing and injecting BMMNC subsets into MI mice as mentioned above, mice were euthanized 7 days after injection. Hearts were carefully removed, embedded into Tissue-Tek OCT (Sakura Finetek, Japan), transversely sectioned into 8 *μ*m slices using a cryostat, and nuclei stained using DAPI. Homing of cells was determined using an OLYMPUS microscope.

### 2.11. Echocardiographic Studies

Heart function was assessed by transthoracic echocardiography on day 7, day 14, and day 21 after MI. Mice were lightly anesthetized using 10% chloral hydrate (0.1 mL/10 g), and chest fur was removed using a depilatory cream. Two-dimensional echocardiographic images and M-mode traces were taken from the parasternal short-axis view at the level of papillary muscles. To evaluate ventricle volume changes, left ventricular end-diastolic volume (EDV) and left ventricular end-systolic volume (ESV) were measured, and ejection fraction (EF) was calculated as an index of systolic function.

### 2.12. Infarct Size Measurement

Infarction size was determined on day 21 after MI. After each mouse was euthanized, the heart was carefully removed, sectioned into ~2 mm transverse sections, and placed in 1% 2,3,5-triphenyltetrazolium chloride (TTC; Sigma-Aldrich, USA) for 30 min at 37°C. We measured the infracted area of each slice using Image J software.

### 2.13. Quantitative Real-Time PCR

Total RNA was isolated from BMMNC subsets and heart tissue using TRIzol Reagent (Takara, Japan) according to manufacturer's instruction. Total RNA (1 *μ*g) was reverse transcribed to cDNA using Reverse Transcription System (Promega) following manufacturer's protocol. Primers for IL-1*β*, IL-6, and TNF-*α* were validated and used in quantitative real-time PCR amplifications. The relative expression levels were then determined using the 2^−ΔΔCt^ method. Primer sequences are shown in Table 1.

### 2.14. ELISA Analyses

Seven days after cell transplantation, mice hearts were collected, rinsed thoroughly with PBS, and homogenized. Homogenates were centrifuged at 3000 rpm for 20 min, and supernatants were collected and stored at −80°C until analysis. IL-1*β*, IL-6, and TNF-*α* were analyzed by ELISA (MultiSciences, China) following manufacturer's instruction.

### 2.15. Statistical Analysis

The results were presented as mean ± SEM. Differences between 2 groups were analyzed by Student's *t*-test. One-way analysis of variance (ANOVA) followed by Tukey analysis was used for multiple comparisons. *p* < 0.05 was considered statistically significant. SPSS 16.0 software was used for statistical analysis.

## 3. Results

### 3.1. AT_2_R Increases in Bone Marrow Mononuclear Cells after MI

**Figure 1:**
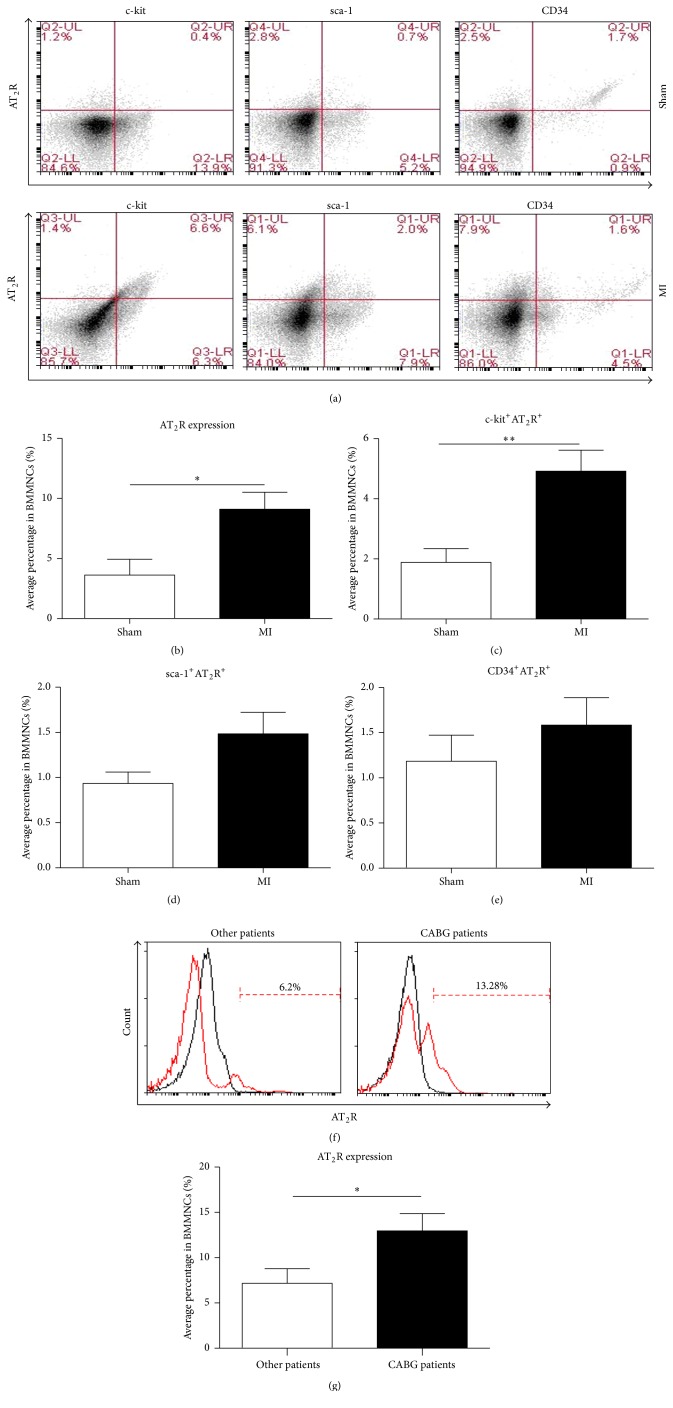
Flow cytometric analysis of BMMNCs. (a) FACS plots of c-kit^+^AT_2_R^+^, sca-1^+^AT_2_R^+^, and CD34^+^AT_2_R^+^ cells isolated from MI and sham mice. (b) FACS plots were quantified as a percentage of AT_2_R^+^ cells in total BMMNCs (^*∗*^ *p* < 0.05, ^*∗∗*^ *p* < 0.01 versus sham; values are means ± SEM; sham, *n* = 6; MI, *n* = 6). ((c)–(e)) Expression analysis of c-kit^+^AT_2_R^+^ (c), sca-1^+^AT_2_R^+^ (d), and CD34^+^AT_2_R^+^ (e) in BMMNCs. Values are means ± SEM (sham, *n* = 6; MI, *n* = 6). (f) FACS plots of AT_2_R^+^ cells isolated from human bone marrow. (g) Statistical expression analysis of AT_2_R^+^ cells in total hBMMNCs (^*∗*^ *p* < 0.05, versus other patients; values are means ± SEM (other patients, *n* = 10; CABG patients, *n* = 10)).

We examined AT_2_R expression in bone marrow mononuclear cells in response to acute ischemic injury. One week after MI, upregulation of cell membrane-associated AT_2_R (mean ± SEM: 9.10 ± 1.41%) was observed in mice. In contrast, AT_2_R was expressed scarcely without MI (mean ± SEM: 3.63 ± 1.31%, *p* < 0.05, Figure 1(b)). Interestingly, we observed the same trend of AT_2_R expression in human BMMNCs; the frequency of AT_2_R^+^ cells in hBMMNC was increased from 7.18 ± 1.61% in controls to 12.96 ± 1.91% in patients who underwent CABG surgery (*p* < 0.05, Figure 1(g)). We also evaluated several stem cell surface markers coexpressed with AT_2_R, including c-kit, sca-1, and CD34 (Figure 1(a)). Notably, AT_2_R was mainly coexpressed with bone marrow c-kit stem cell surface marker in response to acute myocardial infarction (mean ± SEM: 4.92 ± 0.69%, *p* < 0.01, Figure 1(c)). In contrast, only 1.48 ± 0.24% BMMNCs coexpressed AT_2_R with the hematopoietic progenitor marker sca-1 (Figure 1(d)), and 1.58 ± 0.30% BMMNCs coexpressed AT_2_R with the endothelial/hematopoietic progenitor marker CD34 (Figure 1(e)). From sham-operated mice, 1.88 ± 0.46% of BMMNCs coexpressed c-kit and AT_2_R, 1.18 ± 0.28% were CD34^+^AT_2_R^+^, and 0.93 ± 0.13% were sca-1^+^AT_2_R^+^ (*n* = 6 for each marker, Figures 1(c)–1(e)). These data show that AT_2_R was upregulated after MI and can be expressed on stem cells, especially c-kit^+^ cells. These results indicate that AT_2_R and the c-kit^+^AT_2_R^+^ BMMNC subpopulation may play a role after MI.

### 3.2. Apoptosis Rate of BMMNC Subsets under Anoxia and Serum-Free Conditions

**Figure 2:**
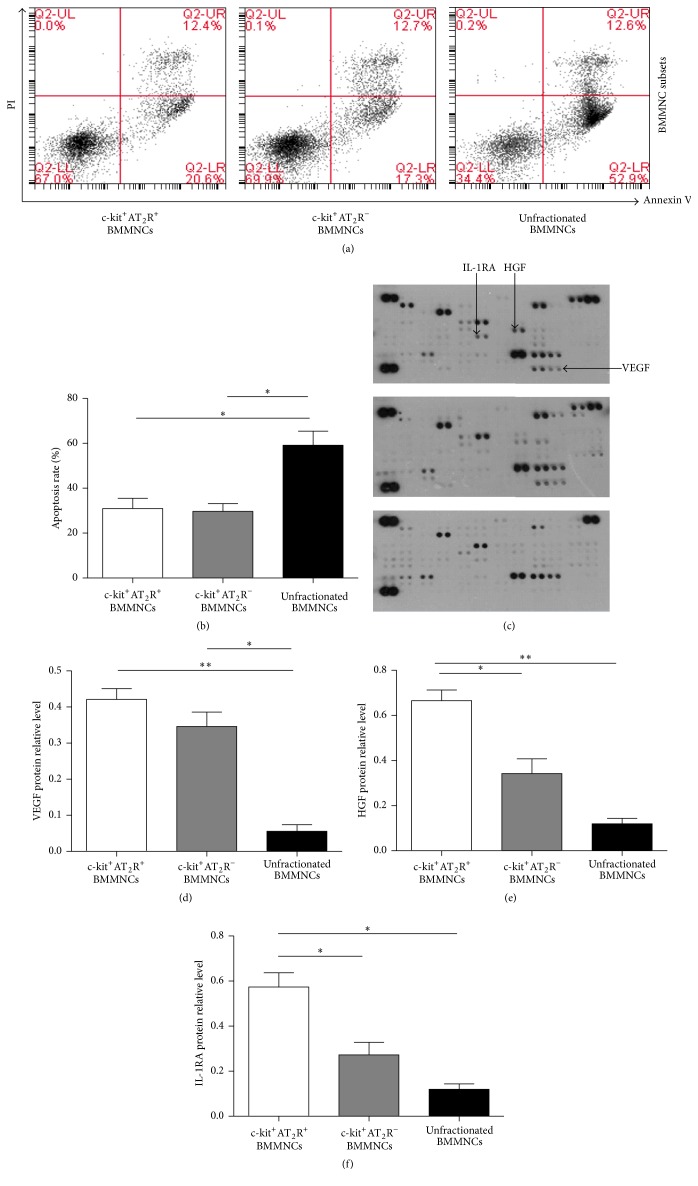
Apoptotic rate of BMMNC subsets. ((a)-(b)) Annexin V/PI staining of BMMNC cells. ^*∗*^ *p* < 0.05; values are means ± SEM; BMMNCs subsets, *n* = 3; ((c)–(f)) Cytokine protein array analysis of VEGF, HGF, and IL-1RA levels in BMMNC subsets. (^*∗*^ *p* < 0.05, ^*∗∗*^ *p* < 0.01; values are means ± SEM).

We aimed to examine the apoptotic rate of these three BMMNC subsets (c-kit^+^AT_2_R^+^, c-kit^+^AT_2_R^−^, and unfractionated BMMNCs) under anoxia and serum-free conditions. This analysis revealed that the c-kit^+^AT_2_R^+^ subpopulation showed 30.97 ± 4.549% apoptotic cells, which was not significantly different from c-kit^+^AT_2_R^−^ subpopulation (mean ± SEM: 29.70 ± 3.466%) but significantly lower than unfractionated BMMNCs (Figures 2(a) and 2(b), mean ± SEM: 59.17 ± 6.253%, *p* < 0.05).

### 3.3. Cytokine Expression of c-kit^+^AT_2_R^+^, c-kit^+^AT_2_R^−^, and Unfractionated Bone Marrow Mononuclear Cells

To further investigate which cytokines were secreted by the different BMMNC subpopulations after exposure to hypoxia in serum-free medium for 48 hours, the supernatant of c-kit^+^AT_2_R^+^ BMMNCs, c-kit^+^AT_2_R^−^ BMMNCs, and unfractionated BMMNCs was collected and analyzed. As shown in Figure 2(c), vascular endothelial growth factor (VEGF) was highly expressed in the supernatant of c-kit^+^AT_2_R^+^ BMMNCs and c-kit^+^AT_2_R^−^ BMMNCs compared to unfractionated BMMNCs (Figure 2(d)). Moreover, c-kit^+^AT_2_R^+^ BMMNCs showed higher amounts of secreted HGF and IL-1 receptor antagonist (RA) proteins than the other two groups (Figures 2(e)-2(f)).

### 3.4. c-kit^+^AT_2_R^+^ Bone Marrow Mononuclear Cells Improve Cardiomyocyte Protective Effects under Hypoxia and Serum-Free Condition* In Vitro*

**Figure 3:**
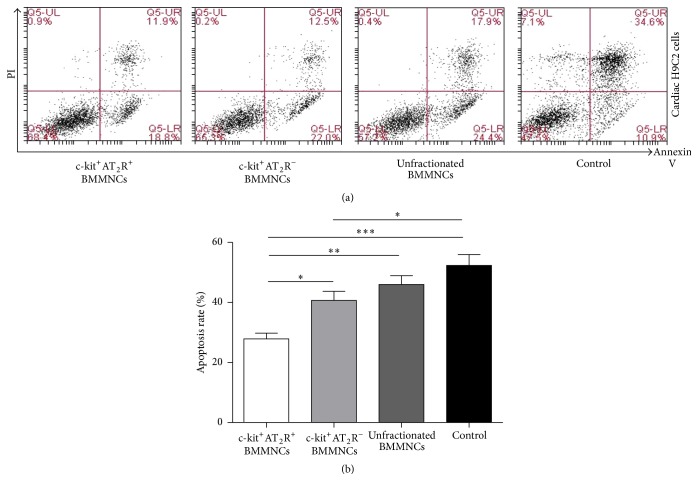
Apoptotic rate of cardiac H9C2 cells. (a) Annexin V/PI staining of cardiac H9C2. (b) Apoptotic rate of cardiac H9C2 cells (^*∗*^ *p* < 0.05, ^*∗∗*^ *p* < 0.01, and ^*∗∗∗*^ *p* < 0.001; values are means ± SEM; BMMNCs subsets, *n* = 6).

Subsequently, we cocultured cardiac H9C2 cells with c-kit^+^AT_2_R^+^, c-kit^+^AT_2_R^−^, or unfractionated BMMNCs, respectively. And exposed the coculture system to hypoxia with serum-free medium for 48 hours. The results showed that the c-kit^+^AT_2_R^+^ BMMNC subpopulation exerted the best protective effect for H9C2 cells against apoptosis (Figures 3(a) and 3(b), mean ± SEM: 27.9 ± 1.86%) compared to c-kit^+^AT_2_R^−^ (Figures 3(a) and 3(b), mean ± SEM: 40.68 ± 3.026%) and unfractionated BMMNC (Figures 3(a) and 3(b), mean ± SEM: 45.98 ± 2.925%) subpopulation. These data confirmed that c-kit^+^AT_2_R^+^ BMMNC subpopulation is superior to c-kit^+^AT_2_R^−^ BMMNCs and unfractionated BMMNCs in protecting against injured cardiomyocytes apoptosis.

### 3.5. Migration Capacity of Bone Marrow Mononuclear Cell Subsets

**Figure 4:**
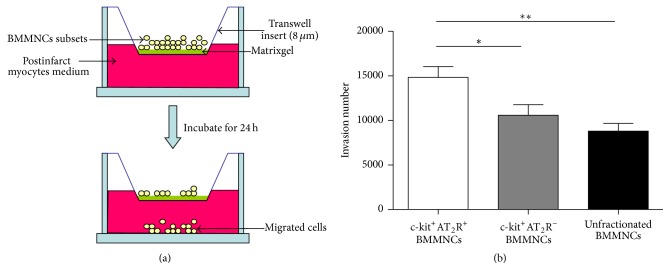
Transwell migration assay of cell subsets derived from mice BMMNCs. (a) A schematic picture of transwell migration assay. (b) Statistical analysis of migrated cell numbers (^*∗*^ *p* < 0.05, ^*∗∗*^ *p* < 0.01; values are means ± SEM; *n* = 5).

Transwell migration assays were utilized to evaluate c-kit^+^AT_2_R^+^, c-kit^+^AT_2_R^−^, and unfractionated BMMNCs motility. A graphical overview is shown in Figure 4(a). Our results showed that c-kit^+^AT_2_R^+^ BMMNCs migrated to the lower chamber more robustly than c-kit^+^AT_2_R^−^ (*p* < 0.05) and unfractionated BMMNCs (*p* < 0.01). Moreover, there was no difference between c-kit^+^AT_2_R^−^ BMMNCs and unfractionated BMMNCs in migration capacity (Figure 4(b), *n* = 6 for each group).

### 3.6. Homing Capacity of Bone Marrow Mononuclear Cell Subsets

**Figure 5:**
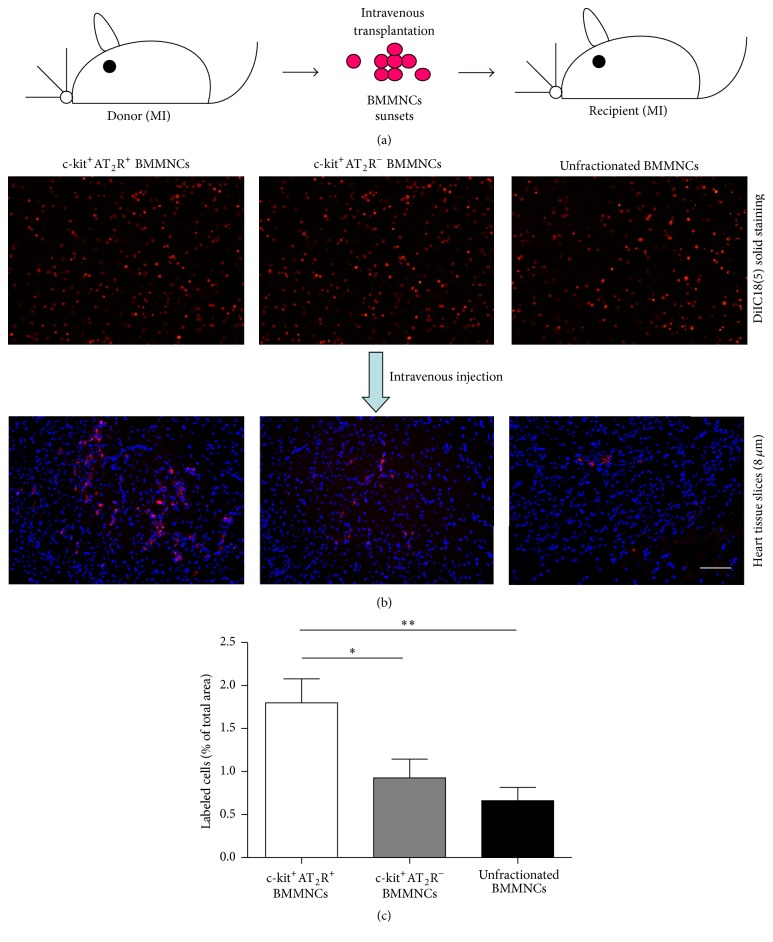
Homing capacity of BMMNC subsets* in vivo*. (a) Transplantation scheme showing BMMNC subsets derived from MI mice transplanted into MI recipients. (b) DiIC18(5) solid staining (red, upper panel; scale bar = 50 *μ*m). Labeled BMMNC subsets were evaluated 7 days after transplantation (lower panel; scale bar = 50 *μ*m). (c) Quantification of homing cells (labeled cells/total area %). ^*∗*^ *p* < 0.05 and ^*∗∗*^ *p* < 0.01.

To confirm that BMMNC subpopulations homing* in vivo* was correlated with* in vitro *results, we further examined the distribution of BMMNCs in the heart of MI mice 1 week after transplantation. The results show that c-kit^+^AT_2_R^+^ BMMNCs migrate more actively into the infarct area than c-kit^+^AT_2_R^−^ and unfractionated BMMNCs (*p* < 0.05, Figures 5(b)-5(c), *n* = 6 for each group), suggesting that AT_2_R may participate in enhancing homing capacity of c-kit^+^AT_2_R^+^ BMMNCs.

### 3.7. Intravenous Transplantation of Bone Marrow Mononuclear Cell Subpopulations Enhanced Cardiac Repair

**Figure 6:**
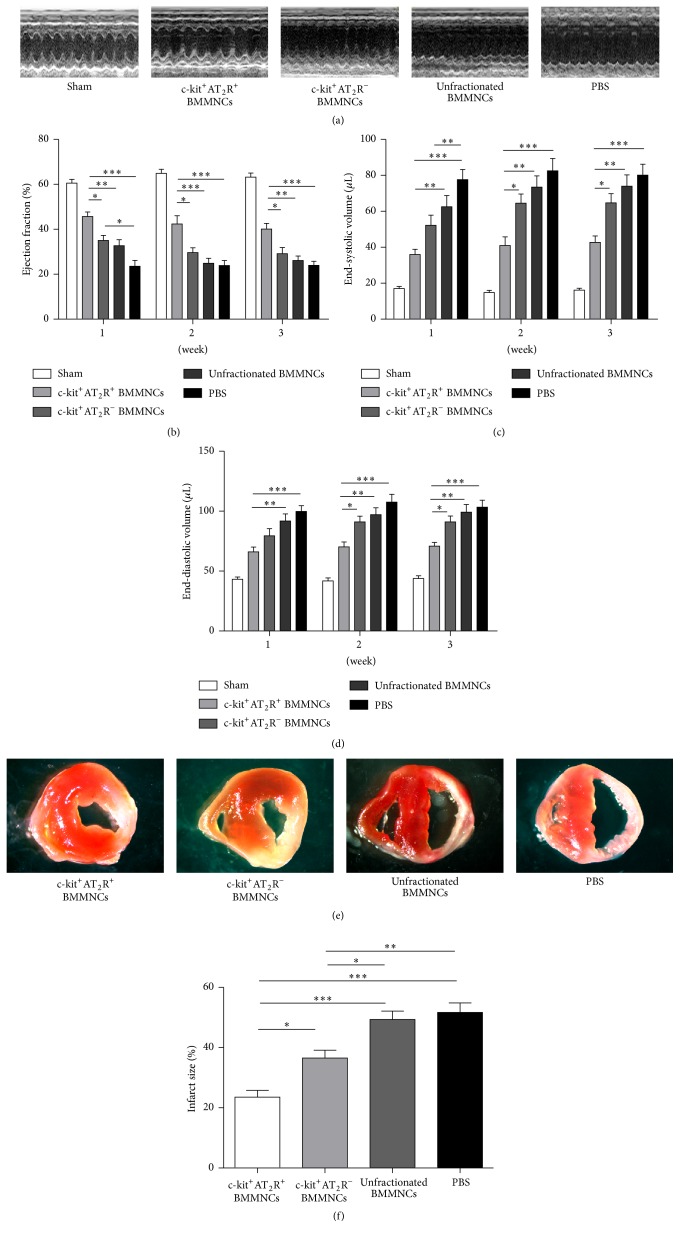
Echocardiographic evaluation: (a) M-mode images showing cardiac function. (b) Ejection fraction, (c) end-systolic volume, and (d) end-diastolic volume (^*∗*^ *p* < 0.05, ^*∗∗*^ *p* < 0.01, and ^*∗∗∗*^ *p* < 0.001; *n* = 6 for each group). (e) TTC staining of transverse sections and quantification of infarct size of each group (^*∗*^ *p* < 0.05, ^*∗∗*^ *p* < 0.01, and *n* = 6 for each group).

To further assess the role of c-kit^+^AT_2_R^+^, c-kit^+^AT_2_R^−^, and unfractionated BMMNCs in cardiac repair, sorted cells were immediately administered to recipient MI mice by using intravenous route (*n* = 6 for each group). Echocardiography examination was performed on day 7, day 14, and day 21 after transplantation. At 1 week following MI, c-kit^+^AT_2_R^+^ BMMNCs group exert a better protection than unfractionated BMMNC and PBS treated groups in end-diastolic volume and end-systolic volume; however, no significant differences were seen between c-kit^+^AT_2_R^+^ and c-kit^+^AT_2_R^−^ group, even though c-kit^+^AT_2_R^+^ group exert a better EF value (Figures 6(b)–6(d)). At 2 and 3 weeks, mice receiving c-kit^+^AT_2_R^+^ BMMNCs had smaller end-systolic volume and end-diastolic volume than the other groups (Figures 6(c)-6(d)), and these reductions were associated with greater EF values in the c-kit^+^AT_2_R^+^ BMMNC injection group (Figure 6(b)). In brief, c-kit^+^AT_2_R^+^ BMMNCs transplantation moderately attenuated but did not completely prevented adverse post-MI left ventricle (LV) function. Moreover, c-kit^+^AT_2_R^−^ and unfractionated BMMNCs transplantations were not as effective as the c-kit^+^AT_2_R^+^ cell population in protecting heart function after MI. The echocardiographic parameters are shown in Table 2.

c-kit^+^AT_2_R^+^ and c-kit^+^AT_2_R^−^ BMMNC subpopulations treatment reduced the infarct size compared with unfractionated BMMNCs and PBS treatment (Figure 6(e)). Moreover, the infarction volumes of mice treated with c-kit^+^AT_2_R^+^ BMMNCs were smaller than c-kit^+^AT_2_R^−^ BMMNCs. These data suggest that c-kit^+^AT_2_R^+^ subset can ameliorate infarction volume better than c-kit^+^AT_2_R^−^ BMMNC subsets after transplantation.

### 3.8. c-kit^+^AT_2_R^+^ BMMNCs Reduced Cardiac Inflammation More Effectively Than c-kit^+^AT_2_R^−^ and Unfractionated Bone Marrow Mononuclear Cells

**Figure 7:**
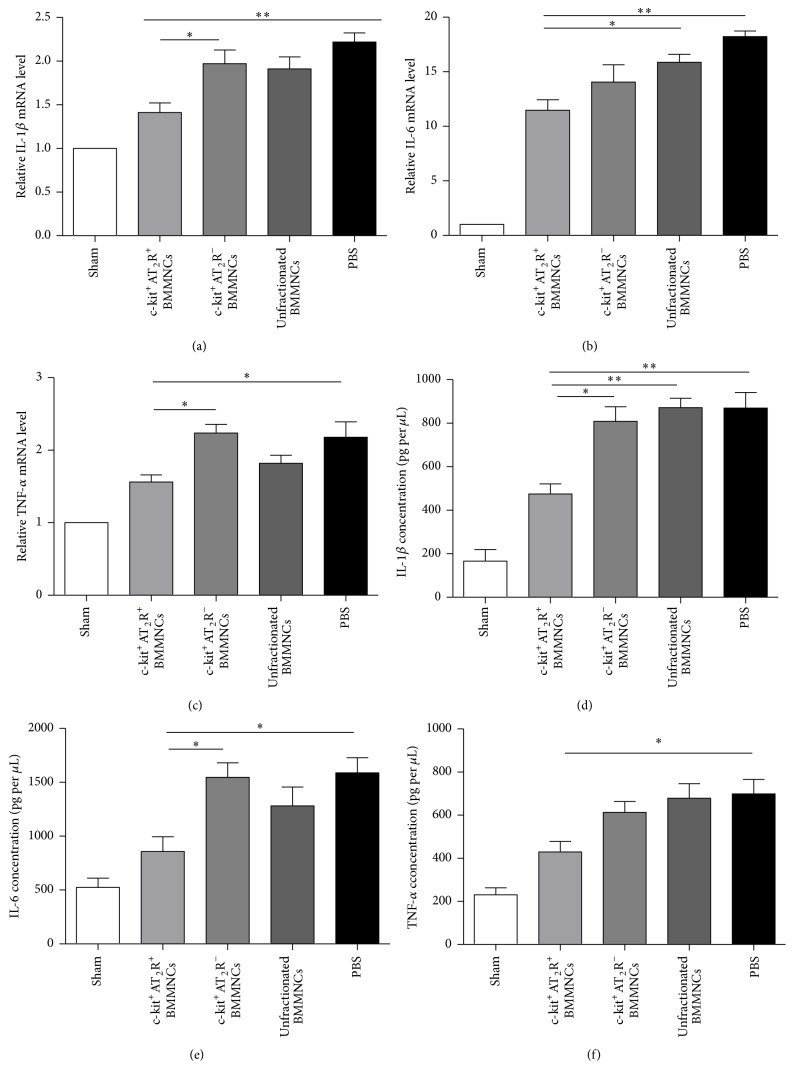
Expression of inflammatory factors in heart tissue. ((a)–(c)) Real-time PCR of IL-1*β*, IL-6, and TNF-*α* mRNA expression level in heart tissues (^*∗*^ *p* < 0.05, ^*∗∗*^ *p* < 0.01; *n* = 3 for each group). ((d)–(f)) protein expression level of IL-1*β*, IL-6, and TNF-*α* in supernatants of heart tissue homogenates (^*∗*^ *p* < 0.05, ^*∗∗*^ *p* < 0.01; *n* = 3 for each group).

Inflammation is well known to exert a great influence on pathology of MI. Inflammatory reactions are a big challenge for implanted cells possibly affecting their survival [18]. Therefore, we examined levels of several proinflammatory factors in the myocardium 7 days after cell injection. The expression of interleukin-1*β* (IL-1*β*), interleukin-6 (IL-6), and tumor necrosis factor-*α* (TNF-*α*) mRNA was less abundant in the myocardium when they are treated with c-kit^+^AT_2_R^+^ BMMNCs compared to the PBS treated groups. Moreover, c-kit^+^AT_2_R^+^ BMMNCs treatment can downregulate IL-1*β* and TNF-*α* mRNA more effectively than c-kit^+^AT_2_R^−^ group (Figures 7(a)–7(c), *n* = 3 for each group). The results of PCR analysis were confirmed by ELISA analysis at protein levels; c-kit^+^AT_2_R^+^ BMMNCs also reduced inflammatory cytokines IL-1*β*, IL-6, and TNF-*α* in the infarcted heart when compared with PBS treated group. Moreover, IL-1*β* and IL-6 were less expressed in c-kit^+^AT_2_R^+^ subset treated group than c-kit^+^AT_2_R^−^ subset group (Figures 7(d)–7(f), *n* = 3 for each group). Thus, outcomes indicated that c-kit^+^AT_2_R^+^ BMMNC transplantation attenuates inflammatory reactions in the post-MI phase of the myocardium.

## 4. Discussion

In this study, bone marrow-derived c-kit^+^AT_2_R^+^, c-kit^+^AT_2_R^−^, and unfractionated BMMNC subpopulations were compared regarding their treatment effects in mice subjected to MI. The major findings are as follows: c-kit^+^AT_2_R^+^ subset is superior to the c-kit^+^AT_2_R^−^ subset in improving cardiomyocyte protection* in vitro*. c-kit^+^AT_2_R^+^ BMMNCs showed a more robust migration capacity than c-kit^+^AT_2_R^−^ and unfractionated BMMNCs* in vitro* and* in vivo*. Compared to c-kit^+^AT_2_R^−^ and unfractionated BMMNCs, intravenous transplantation of c-kit^+^AT_2_R^+^ BMMNC resulted in smaller infarct size and lower levels of inflammatory reactions in heart tissue. c-kit^+^AT_2_R^+^ BMMNC subset is superior to c-kit^+^AT_2_R^−^ subset in ameliorating myocardial damage and left ventricle remodeling. Summarized, our data raise the possibility that expansion and myocardial transplantation of autologous c-kit^+^AT_2_R^+^ BMMNCs may be a promising therapeutic approach in post-MI treatment.

Currently, cell therapy using BM-derived stem cells has sparked intense hope considering cardiac repair. Cumulative animal studies have revealed that BMMNC transplantation can improve heart function following MI [19, 20]. We report for the first time AT_2_R expression on c-kit^+^ BMMNCs and c-kit^+^AT_2_R^+^ BMMNC subpopulation increased in response to MI in mice. We also investigated the AT_2_R expression in patients BMMNCs who underwent cardiac artery bypass graft (CABG) surgery and those who had no ischemic heart disease. Consequently, we observed a higher expression of AT_2_R in patients who underwent CABG surgery than in nonischemic donors. AT_2_R is highly expressed in fetal tissues, but its expression dramatically decreases after birth [15, 16]. However, reexpression of AT_2_R, which has been observed in the ischemic heart after acute myocardial infarction, demonstrated the hypothesis that AT_2_R may play an important role in the pathophysiology of MI [17]. It has been indicated that cardiovascular diseases such as MI may significantly impair the functional activity of endogenous stem cells and reduce the efficacy of implanted cells for therapeutic use [21–23], which partly explains why the transplanted c-kit^+^AT_2_R^−^ subpopulation was not so efficient as expected on homing capacity and antiapoptosis in our study. Interestingly, we discovered that c-kit^+^ cells which coexpress AT_2_R exert a better protective function compared to c-kit^+^AT_2_R^−^ cells, suggesting that AT_2_R may partly ameliorate the functional impairment of c-kit^+^ BMMNCs.

It is well established that cardiomyocyte apoptosis remains a major contributor to the loss of myocardium and impaired pump function of the heart [24]. Previous work showed that using ARB (AT_1_R blocker) to stimulate BMMNCs mediates cardiac repair, providing evidence that blocking AT_1_R or, thus, indirectly stimulating AT_2_R may contribute to the cardiac regenerative process [4, 7]. Of note, directly stimulating AT_2_R with C21 reduced the elevated expression of apoptotic markers Fas-ligand and caspase-3 in the infarct border zone. Furthermore, the rescue of mitogen activated protein kinase is also involved in AT_2_R-mediated antiapoptotic effects [18]. Although there is no statistic difference in apoptosis rate between c-kit^+^AT_2_R^+^ and c-kit^+^AT_2_R^−^ BMMNCs cultured under hypoxia and serum-free condition, interestingly, we discovered that the c-kit^+^AT_2_R^+^ subset secretes high levels of VEGF and HGF under anoxia and serum-free condition. Of note, VEGF has demonstrated beneficial effects on angiogenesis by decreasing apoptosis and improving heart functions [25]. Additionally, hepatocyte growth factor (HGF) also has been shown to exert antifibrotic, proangiogenic, and cardioprotective effects [26]. Our* in vitro* study also showed that coculture using investigated BMMNC subpopulations can protect cardiac H9C2 cells from apoptosis induced by hypoxia and serum-free stress and finally revealed that the c-kit^+^AT_2_R^+^ subset exerts the best protective effect compared to the other subsets. Thus, we hypothesize that the c-kit^+^AT_2_R^+^ BMMNC subpopulation participates in the cardioprotective process mainly due to the secretion of VEGF and HGF. Although c-kit^+^AT_2_R^−^ BMMNCs also secrete higher level of VEGF compared to unfractionated BMMNCs, c-kit^+^AT_2_R^+^ subset still exerts a better protection. Thus, it is tempting to infer that AT_2_R enhances the secretion of c-kit^+^ BMMNCs. In summary, our data show that the c-kit^+^AT_2_R^+^ BMMNC subpopulation is superior to c-kit^+^AT_2_R^−^ BMMNCs and unfractionated BMMNCs in protecting against injured cardiomyocytes apoptosis and also suggest that AT_2_R signaling may promote paracrine actions of c-kit^+^ BMMNCs which support survival of cardiac H9C2 cells.

AT_2_R stimulation has been demonstrated to increase c-kit^+^AT_2_R^+^ cell population in the infarcted myocardium. However, the possible origin of the increased number of c-kit^+^AT_2_R^+^ cells in the heart remains somewhat unsettled. One of the key findings of our study is the early increase of c-kit^+^AT_2_R^+^ cell numbers in the bone marrow of MI mice, and we assume that BMMNC subpopulations reside and proliferate in bone marrow upon receiving stimulatory signals from injured tissues like myocardium and are subsequently released into the circulatory system. Furthermore, AT_2_R may help BMMNCs target infarcted myocardial tissue. Thus, we further compared the migration and invasion capacity of c-kit^+^AT_2_R^+^, c-kit^+^AT_2_R^−^, and unfractionated BMMNCs* in vitro* and* in vivo*. The results confirmed our hypothesis that c-kit^+^AT_2_R^+^ subpopulation migrates more actively into the infarct area than c-kit^+^AT_2_R^−^ and unfractionated BMMNCs.

One of the inspiring findings in our study is that the substantial augmentation of circulatory c-kit^+^AT_2_R^+^ cell numbers by intravenous cell transplantation at the time of coronary ligation does attenuate structural and functional remodeling following 1, 2, and 3 weeks. Our findings indicate one possible mechanism: grafted c-kit^+^AT_2_R^+^ BMMNCs may be able to migrate to the damaged heart and infiltrate the infarct area where they release anti-inflammatory and antiapoptotic cytokines such as IL-1RA, HGF, and VEGF to support the survival of residual myocardium.

There is clear evidence showing that an inflammatory reaction can severely augment postinfarct cardiac damages and reject implanted cells, thereby affecting their plasticity and long-term survival [27, 28]. Moreover, several studies showed that c-kit might be involved in the inflammatory process and thus limited the use of c-kit^+^ stem cells for cell therapy [8, 9]. Thus, repressing the immune reaction following MI is a key for enhancing the survival of implanted cells. Although data about the role of the AT_2_R in inflammation are still rather sparse, they support anti-inflammatory features in the majority of cases [29, 30]. Moreover, Curato et al. identified the noncytotoxic CD8^+^ AT_2_R^+^ T cell population, which mediates anti-inflammatory and cardioprotective actions against ischemic heart injury [31]. In our study, we found that the myocardium from c-kit^+^AT_2_R^+^ BMMNC treated group shows lower levels of proinflammatory IL-1*β*, IL-6, and TNF-*α* compared to c-kit^+^AT_2_R^−^ group. This observation may be partly related to the anti-inflammatory protein IL-1RA which is produced by c-kit^+^AT_2_R^+^ BMMNC subset and we here report for the first time that AT_2_R should be responsible at least in part for the repressive effect on inflammatory reaction caused by c-kit^+^ cells. This finding may promote a broader use of c-kit^+^ stem cells in cell therapy.

In summary, we have identified a bone marrow c-kit^+^AT_2_R^+^ cell population that increases in response to ischemic cardiac injury.* In vitro*, we showed that c-kit^+^AT_2_R^+^ cells are superior to c-kit^+^AT_2_R^−^ and unfractionated BMMNCs in antiapoptosis and migration capacity. Furthermore, we provide evidence that intravenous implanted c-kit^+^AT_2_R^+^ BMMNCs are able to infiltrate the heart and are superior to c-kit^+^AT_2_R^−^ and unfractionated BMMNCs in supporting cardiomyocyte survival and repressing cardiac inflammation, ultimately improving global heart function and reducing infarct size in the MI mice model. In conclusion, our findings suggest that AT_2_R partly ameliorates the c-kit^+^ bone marrow precursor cell functions in supporting cardiomyocyte performance in response to MI and, thus, may provide a new strategy to improve stem cell therapeutic effects.

## Figures and Tables

**Table 1:** Primers for qRT-PCR.

Gene	Primer sequence
GAPDH	F: AGTCCCTGCCCTTTGTACACA
	R: CCGAGGGCCTCACTAAACC

IL-1*β*	F: AGGCTTCCTTGTGCAAGTGT
	R: TGAGTGACACTGCCTTCCTG

IL-6	F: CCGGAGAGGAGACTTCACAG
	R: ACAGTGCATCATCGCTGTTC

TNF-*α*	F: GGCTGCCCCGACTACGT
	R: AGGTTGACTTTCTCCTGGTATGAGA

All sequences are in 5′-3′ direction.

**Table 2:** Echocardiography examination.

	EF value (%)			End-systolic volume (*µ*L)			End-diastolic volume (*µ*L)		
	1 week	2 weeks	3 weeks	1 week	2 weeks	3 weeks	1 week	2 weeks	3 weeks
Sham	60.55 ± 4.01	64.8 ± 4.50	63.2 ± 4.37	17.6 ± 2.73	14.75 ± 3.13	16.11 ± 2.66	43.1 ± 4.62	41.78 ± 6.09	43.87 ± 5.29
c-kit^+^AT_2_R^+^ BMMNC	45.6 ± 4.93	42.3 ± 9.01	40.1 ± 6.15	35.9 ± 7.12	41.00 ± 11.64	42.75 ± 8.66	66.0 ± 9.75	70.82 ± 9.89	70.83 ± 7.67
c-kit^+^AT_2_R^−^ BMMNC	35.0 ± 5.69	29.6 ± 5.33	29.1 ± 6.62	52.01 ± 14.03	64.56 ± 12.47	64.70 ± 12.55	79.43 ± 14.80	91.10 ± 11.49	91.02 ± 11.99
Unfractionated BMMNCs	32.6 ± 6.59	24.9 ± 5.28	26.0 ± 4.94	62.53 ± 15.24	73.45 ± 15.32	73.93 ± 15.61	91.78 ± 14.37	97.08 ± 14.07	99.22 ± 15.40
PBS	23.5 ± 6.33	23.9 ± 5.31	23.5 ± 4.43	77.66 ± 13.84	82.51 ± 16.91	80.11 ± 14.93	99.82 ± 11.52	107.6 ± 15.75	103.3 ± 14.25

EF: ejection fraction. *n* = 6 for each group; values are means ± SEM.
